# Resignation of Prof. Walter Channing

**Published:** 1854-10

**Authors:** 


*Resignation of Prof. Walter Channing.*—We learn from the Boston Medical and Surgical Journal, that Prof. Channing, so long known as an eminent teacher of Midwifery and Medical Jurisprudence in the Medical Department of Harvard University, has resigned his chair.

Dr. D. Humphreys Storers has been appointed to fill the vacancy. Dr. Storers' name is a familiar one to the medical public, and will not weaken the strength of the school.

The resignation of Dr. Channing recalls to our mind a very clever pun once perpetrated by him. A gentleman, evidently of the clerical profession, called, and, inquiring for Dr. Channing, was ushered into the presence of the distinguished physician. The Dr. soon perceived from the clerical character of his visitor's remarks, that he labored under the mistaken idea that he was conversing with the equally distinguished *divine*, Dr. Channing.

He at once corrected the error by saying "O! sir! It is my brother William that you wish to see. *He* is the Dr. Channing that *preaches; I practice!*"

